# Cortical Hemodynamic Abnormalities Associated With Fine Motor Deficits in Mild Cognitive Impairment

**DOI:** 10.1111/cns.70547

**Published:** 2025-07-28

**Authors:** Han Yang, Jing Teng, Yilun Qian, Taicheng Huang, Manyu Dong, Huanping Wang, Jie Song, Yuxuan Zhang, Mingming Zhang, Hanjun Liu, Ying Shen

**Affiliations:** ^1^ Department of Rehabilitation Medicine, The First Affiliated Hospital with Nanjing Medical University Nanjing China; ^2^ Shanghai Key Laboratory of Psychotic Disorders, Brain Health Institute, National Center for Mental Disorders, Shanghai Mental Health Center Shanghai Jiao Tong University School of Medicine Shanghai China; ^3^ School of Psychology Shanghai Jiao Tong University Shanghai China; ^4^ School of Psychology Shanghai Normal University Shanghai China; ^5^ Department of Rehabilitation Medicine The First Affiliated Hospital, Sun Yat‐Sen University Guangzhou China; ^6^ Guangdong Provincial Key Laboratory of Brain Function and Disease, Zhongshan School of Medicine Sun Yat‐Sen University Guangzhou China

**Keywords:** cortical hemodynamic response, fine motor deficits, function near‐infrared spectroscopy, mild cognitive impairment

## Abstract

**Background:**

Individuals with mild cognitive impairment (MCI) often exhibit progressive deficits in bimanual coordination and fine motor dexterity. However, the neural mechanisms underlying these motor impairments remain poorly understood.

**Aim:**

This cross‐sectional study employed functional near‐infrared spectroscopy (fNIRS) to examine cortical hemodynamic responses during fine motor tasks in MCI.

**Methods:**

Thirty individuals with MCI and 40 age‐, sex‐, and education‐matched healthy controls (HCs) performed the Nine‐Hole Peg Test (NHPT) while fNIRS monitored oxygenated hemoglobin (HbO) and deoxygenated hemoglobin (HbR) responses in the prefrontal cortex (PFC), sensorimotor cortex (SMC), and visual cortex (VC).

**Results:**

Compared to HCs, individuals with MCI exhibited significantly impaired NHPT performance, accompanied by reduced HbO responses in the right PFC and SMC during task performance. Furthermore, stepwise discriminant analysis identified a combination of right SMC HbO levels and NHPT scores as a significant predictor for distinguishing MCI from HCs, achieving an area under the curve (AUC) of 80.8%.

**Conclusions:**

These findings provide novel evidence linking aberrant cortical hypoactivation in the motor and executive control regions to fine motor impairments in individuals with MCI, suggesting disrupted motor‐cognitive integration in early cognitive decline. The integration of fNIRS‐derived hemodynamic responses with functional motor assessments offers a promising non‐invasive approach for MCI detection and personalized rehabilitation.

**Trial Registration:**

This study was registered with the Chinese Clinical Trial Registry (Registration No. ChiCTR2400082429) on March 28, 2024

## Graphical abstract

Compared to HCs, individuals with MCI exhibited significantly impaired NHPT performance, accompanied by reduced HbO responses in the right PFC and SMC during task performance. A combination of right SMC HbO levels and NHPT scores served as a significant predictor for distinguishing MCI from HCs, achieving an AUC of 80.8%.

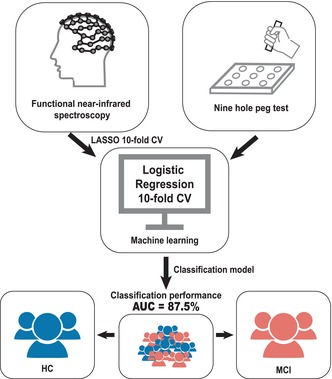

## 1 Introduction

Over the past several decades, the global population has experienced a rapid aging process, accompanied by an increase in health‐adjusted life expectancy (HALE), which estimates the average number of years a person can expect to live in good health without significant disease or disability, rising from 61.3 years (95% UI: 58.6–63.6) in 2010 to 62.2 years (95% UI: 59.4–64.7) in 2021 [1]. This demographic shift has heightened a growing prevalence of age‐related cognitive disorders, including mild cognitive impairment (MCI) and dementia [2, 3]. MCI is widely recognized as an intermediate stage between normal aging and dementia, characterized by the presence of self‐ or informant‐reported cognitive complaints, objective evidence of cognitive decline on standardized assessments, and preservation of functional independence without manifest dementia [4, 5]. Early symptoms of MCI include memory impairment, executive dysfunction, and visuospatial deficits, which can progressively worsen and lead to dementia [6]. A recent meta‐analysis reported an overall prevalence of MCI at 15.56% among community‐dwelling adults over the age of 50 years worldwide [7]. Moreover, 10%–15% of individuals with MCI progress to Alzheimer's disease (AD) annually [8]. These findings highlight the urgent need for early detection and intervention strategies to mitigate cognitive decline in aging populations.

To date, the diagnosis of MCI and dementia relies primarily on standardized neuropsychological tests and clinical interviews. However, considerable efforts have been dedicated to identifying biomarkers capable of detecting the preclinical stage of dementia [9]. In 2024, the National Institute on Aging and the Alzheimer's Association updated diagnostic guidelines, emphasizing that AD should be defined biologically rather than solely based on clinical syndromes [10]. In recent years, a variety of biomarkers and neuroimaging techniques have been employed to evaluate risk factors for AD. For example, cerebrospinal fluid (CSF) SNAP‐25 has been identified as a highly specific biomarker, exhibiting excellent diagnostic accuracy even in early disease stages [11]. Also, blood phosphorylated tau (p‐tau181) has shown promise in predicting tau and amyloid β pathologies, distinguishing AD from other neurodegenerative disorders [12]. In addition, tau amyloid positron emission tomography (PET) has demonstrated high sensitivity and specificity in predicting cognitive decline in individuals with MCI [13]. Despite these advances, clinical applications of these biomarkers remain limited due to the invasiveness of CSF extraction, the technical complexity of blood p‐tau181 analyses, and the high cost of PET imaging.

Emerging evidence suggests that both fine and gross motor impairments, such as gait changes associated with motor‐cognitive risk (MCR) syndrome, may serve as early markers of cognitive decline and dementia risk [14, 15, 16, 17, 18, 19]. Among fine motor domains, deterioration in manual dexterity may offer a potential window for the identification of individuals at heightened risk of cognitive deterioration [20]. Individuals with MCI, particularly across different subtypes, exhibit more pronounced bradykinesia (slowness of movement) and reduced motor dexterity in both upper and lower limbs compared to healthy controls (HCs) [21]. Furthermore, kinematic studies have shown that fine motor control in individuals with MCI is characterized by slower, less smooth, less coordinated, and less consistent movements compared to HCs [22, 23]. These findings suggest fine motor tasks may hold the potential for distinguishing between individuals with MCI and HCs.

Nevertheless, previous research has primarily focused on behavioral assessments of fine motor skills among individuals with MCI, leaving the neural mechanisms underlying these motor deficits poorly understood. Functional near‐infrared spectroscopy (fNIRS), an optical neuroimaging technique based on neurovascular coupling, offers a non‐invasive approach to assess cortical hemodynamic responses by measuring changes in oxygenated (HbO) and deoxygenated hemoglobin (HbR) concentrations [24]. Compared to functional magnetic resonance imaging (fMRI) and electroencephalography (EEG), fNIRS exhibits greater tolerance to motion artifacts and has reasonable spatial resolution, making it particularly suitable for monitoring cortical activation during fine motor tasks. Importantly, fNIRS has shown significant promise in identifying oxygenation‐based biomarkers for cognitive assessment, distinguishing healthy aging, MCI, and dementia (including AD) [25, 26, 27, 28]. Distinct cortical perfusion and oxygenation patterns between MCI and dementia have been observed during tasks such as working memory, phonemic fluency, and motor activities [25]. Furthermore, individuals with MCI exhibited disruptions in fNIRS‐based functional connectivity (FC) compared to HCs during both resting states and cognitive tasks (e.g., working memory and verbal fluency) [28]. In addition, fNIRS has provided insights into compensatory mechanisms during fine motor tasks. For example, patients with Parkinson's disease (PD) exhibited delayed hypoactivation in the motor cortex of the dominant hand during the motor task [29], while individuals with subjective cognitive decline (SCD) showed increased prefrontal activation during cognitive‐motor dual tasks [30]. These findings highlight the potential of fNIRS as a sensitive, non‐invasive tool for detecting cortical alterations associated with fine motor impairments in individuals with MCI.

To this end, the present study employed fNIRS to investigate cortical hemodynamic patterns during fine motor task performance in individuals with MCI, shedding light on the potential differences in neural correlates of fine motor control between individuals with MCI and healthy controls. Fine motor skills were assessed using the Nine‐Hole Peg Test (NHPT), a widely used measure of manual dexterity [31] that has demonstrated excellent validity and intra‐rater reliability across both healthy individuals and patients with neurological disorders [32, 33]. Both HbO and HbR responses were used to assess task‐related cortical activation during the NHPT. Based on previous findings of altered cortical activation during cognitive and motor tasks in MCI, we hypothesized that individuals with MCI would exhibit distinct cortical hemodynamic responses compared to HCs, particularly within prefrontal and motor‐related regions.

## 2 Methods

### 2.1 Study Design and Participants

The cross‐sectional study protocol adhered to the ethical standards outlined in the Declaration of Helsinki and was approved by the Ethics Committee of the First Affiliated Hospital with Nanjing Medical University (No. 2023‐SR‐792). This study was registered with the Chinese Clinical Trial Registry (Registration No. ChiCTR2400082429) on March 28, 2024. A total of 30 individuals with MCI and 40 HCs, aged between 53 and 79 years (MCI: 66.90 ± 6.76 years; HCs: 64.20 ± 6.95 years), were recruited. Participants in both groups were matched for age, sex, and years of education. Individuals with MCI were recruited from the First Affiliated Hospital with Nanjing Medical University and were diagnosed by a psychiatrist based on comprehensive neuropsychological assessments. All participants received a detailed explanation of the study procedure and provided written informed consent prior to participation.

Participants were excluded if they met any of the following conditions: (1) younger than 50 or older than 80 years; (2) diagnosis of vascular dementia or dementia based on the National Institute of Neurological Disorders and Stroke–Association Internationale pour la Recherche et l'Enseignement en Neurosciences (NINDS‐AIREN) criteria [34]; (3) a Modified Hachinski Ischemic Score > 4 [35]; (4) a Geriatric Depression Scale score ≥ 6 [36]; (5) a history of drug or alcohol dependence within the past 6 months; (6) inability to complete neuropsychological assessments due to cognitive or physical limitation; (7) presence of severe cerebrovascular or psychiatric disorders; (8) severe scalp injuries, open head wounds, or intolerance to wearing headgear; (9) use of antidepressants, anxiolytics, or central nervous system medications that could affect cortical excitability within 3 months prior to assessment; (10) non‐right‐handedness determined using the Edinburgh Handedness Inventory [37].

Participants were included in the MCI group if they met any one of the following three criteria: [38, 39] (1) performance ≥ 1 standard deviation (SD) below the age‐adjusted normative mean on both assessments within at least one cognitive domain (memory, language, or executive function); or (2) performance ≥ 1 SD below the age‐adjusted normative mean on one assessment in each of the three cognitive domains (memory, language, or executive function); or (3) a Functional Activities Questionnaire (FAQ) score ≥ 9 [40]. The SD‐based cutoffs were derived from established normative data on the Chinese population, adjusted for age and education [41]. The inclusion criteria for the HC group included: (1) no self‐ or informant‐reported memory decline; (2) a Mini‐Mental State Examination (MMSE) score > 26 [42]; (3) a Montreal Cognitive Assessment (MoCA) score > 26 [43]; (4) a Clinical Dementia Rating (CDR) score of 0 [44].

### 2.2 Sample Size

Previous research reported a SD of approximately 0.8 for the effect size associated with the NHPT performance [45]. A two‐tailed test was implemented with an α level of 0.05, and the allowable error was defined as 10% of the mean, corresponding to a value of −0.3. Using G*Power software [46], the minimum required sample size was calculated to be 28 participants per group. To account for potential data loss or invalid measurements (~5%), the final sample size was increased to 30 participants per group. In practice, 40 participants were enrolled in the control group to enhance statistical power and improve data stability.

### 2.3 Nine‐Hole Peg Test

All participants were assessed using NHPT as the primary measure of fine motor movement [32]. During the task, participants were seated with table and chair height adjusted to ensure their comfort. To prevent movement of the apparatus and minimize potential variations in task performance, a non‐slip foam sheet was placed beneath the pegboard. Participants used their left hand to stabilize the board while performing the task with their right hand. Following instructions, they inserted and removed as many pegs as possible within a 30‐s trial, followed by a 30‐s rest period. Each task‐rest cycle constituted one block, with the rest blocks serving as baseline periods for evaluating task‐related hemodynamic changes. This process was repeated for three blocks, and the number of pegs inserted and removed was recorded as the primary performance metric [47, 48, 49].

### 2.4 Neuropsychological Assessment

Cognitive function was comprehensively evaluated using standardized neuropsychological tests. Global cognitive function was assessed using the MoCA and MMSE. Memory function was evaluated using the Auditory Verbal Learning Test‐Huashan Version (AVLT‐H) [50]. Language function was assessed using the Boston Naming Test‐Chinese Version (BNT‐C) and the Animal Fluency Test (AFT) [51, 52]. Executive function was evaluated using the FAQ, the Shape Trial Test‐A Chinese Version (STT‐A‐C), and the Shape Trial Test‐B Chinese Version (STT‐B‐C) [53].

### 2.5 fNIRS Measurement

**FIGURE 1:**
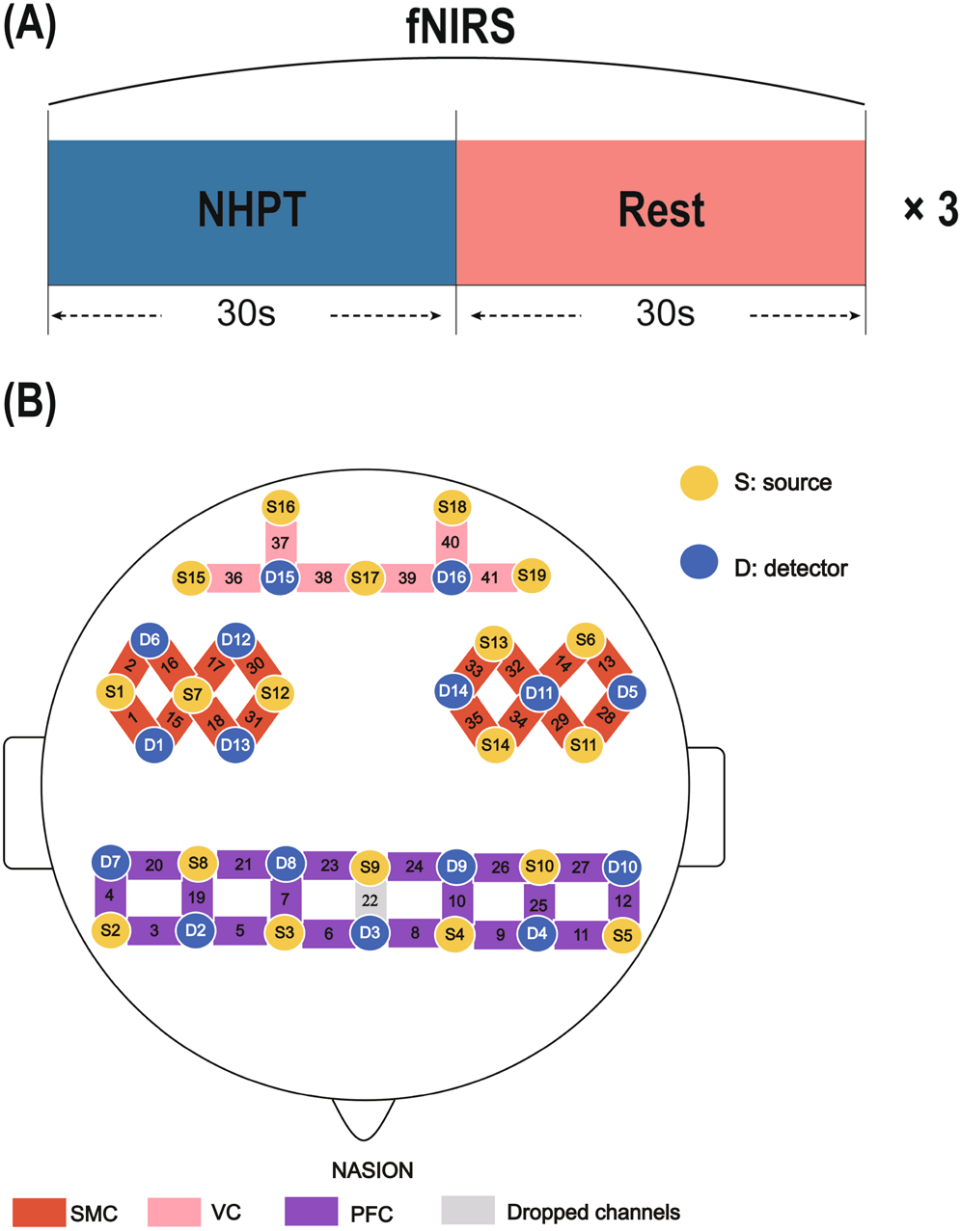
(A) Schematic of the fNIRS testing procedure for individuals with MCI and HCs during the NHPT. (B) fNIRS probe and channel mapping illustrating the spatial arrangement of transmitting probes (yellow) and receiving probes (blue), positioned to cover three regions of interest (ROIs): SMC (red), PFC (purple), and VC (pink). The Nasion landmark was used to standardize probe alignment across participants. Channels are numbered according to their corresponding measurement locations within each ROI. Abbreviations: FNIRS, functional near‐infrared spectroscopy; HCs, healthy controls; MCI, mild cognitive impairment; NHPT, Nine‐Hole Peg Test; PFC, prefrontal cortex; ROIs, regions of interest; SMC, sensorimotor cortex; VC, visual cortex.

Both individuals with MCI and HCs underwent fNIRS recording to monitor cortical hemodynamic responses during the NHPT task (Figure 1A). The NirSmart‐6000A system (Danyang Huichuang Medical Equipment Co. Ltd., China) was used to record changes in HbO and HbR concentration throughout the task. The system consists of near‐infrared light sources (LEDs) and avalanche photodiodes (APDs) as detectors, with wavelengths of 730 nm and 850 nm and a sampling rate of 11 Hz. The experimental setup consisted of 19 light sources and 16 detectors, forming a total of 41 measurement channels with an average source‐detector distance of 3 cm (range 2.7–3.3 cm) (Table S1). Anatomical localization of optodes and channels was performed using a standardized three‐dimensional (3D) head model, with cranial landmarks including the nasion (Nz), inion (Iz), left preauricular point (AL), right preauricular point (AR), and vertex (Cz) as references [54]. The scalp positions were then projected onto a three‐dimensional reconstruction of the cortical surface. Subsequently, the MNI coordinates with maximum likelihood estimation and the corresponding anatomical locations for each channel were obtained using NIRS‐SPM software [55, 56]. This study focused on cortical activation within three regions of interest (ROIs): the bilateral sensorimotor cortex (SMC), prefrontal cortex (PFC), and visual cortex (VC) (see Figure 1B; Table S2).

### 2.6 fNIRS Data Preprocessing

The raw fNIRS data were preprocessed using HomER2 (https://www.nitrc.org/projects/homer2) [57]. First, bad channels were identified through visual inspection, and data with more than 11 bad channels (25% of total channels) were excluded from further analysis. The remaining intensity signals were then converted into changes in optical density (OD) using the hmrIntensity2OD.m function. Motion artifacts were identified using the hmrMotionArtifactByChannel.m function with the following parameters: tMotion = 0.5, tMask = 1, STDEVthresh = 50, and AMPthresh = 0.5. Principal component analysis (PCA) filtering was then applied to remove motion‐ and systemic physiology‐related artifacts using the hmrMotionCorrectPCA.m function with the number of components (nSV) set to 0.8 [58, 59], as done in previous work [60]. Following motion correction, the OD data were band‐pass filtered between 0.01 and 0.09 Hz with the hmrBandpassFilt.m function to remove low‐frequency drifts and physiological oscillations [61, 62]. Finally, changes in HbO and HbR concentrations were calculated from the filtered OD data using the hmrOD2Conc.m function with a differential pathlength factor of 6 for both wavelengths (Table S3). Following preprocessing, HbO and HbR concentration data were extracted separately for each task and rest period, then averaged across trials for subsequent statistical analysis.

It is noteworthy that HbO signals were selected as the primary measure for statistical analysis in the present study. Compared to HbR signals, HbO signals typically exhibit larger amplitude changes and higher signal‐to‐noise ratios, making them more sensitive for detecting task‐related cortical activation [57]. Moreover, HbO signals have been shown to correlate more consistently with fMRI BOLD signals compared to HbR signals [63]. Given these advantages, and in line with prior fNIRS research focused on fine motor tasks [64], we prioritized the use of HbO signals to optimize sensitivity for detecting group difference. Nevertheless, to provide a comprehensive overview of cortical hemodynamic responses, we also analyzed the HbR data following identical preprocessing methods. The results of the HbR analyses are presented in Tables S4–S6.

### 2.7 Statistical Analysis

Statistical analyses were conducted to examine differences between the MCI and HC groups across demographic characteristics, neuropsychological assessments, and fNIRS‐derived hemodynamic responses. The Shapiro–Wilk test was applied to assess the normality of all continuous variables prior to further analyses. Normally distributed variables were reported as mean ± SD, whereas non‐normally distributed or ranked variables were presented as median (interquartile ranges, IQRs). Baseline comparisons between groups were performed using independent 2‐sample t tests for normally distributed continuous variables, Mann–Whitney U tests for non‐normally distributed continuous variables, and *χ* ^2^ tests for categorical variables. Within‐group differences in HbO/HbR concentration between task and rest periods were analyzed using paired t tests. Between‐group differences in HbO/HbR concentration were assessed using analysis of covariance (ANCOVA), adjusting for age, sex, and years of education as covariates.

To explore the clinical potential of our findings, we further sought to establish a predictive model based on cortical hemodynamic responses. Specifically, we aimed to determine whether fNIRS‐derived features, in combination with fine motor performance, could effectively discriminate individuals with MCI from HCs. To refine feature selection, least absolute shrinkage and selection operator (LASSO) regression with 10‐fold cross‐validation was applied to the HbO features identified through ANCOVA. The optimal Lambda.lse value was selected to balance model performance and minimize the number of independent variables. A logistic regression model with 10‐fold cross‐validation was then constructed using the features selected via LASSO regression, along with additional variables of interest. To improve prediction accuracy, the model was adjusted for covariates including age, sex, and education level. Model fit was assessed using the Hosmer–Lemeshow goodness‐of‐fit test.

To control for multiple comparisons, the false discovery rate (FDR) correction was applied with a significance threshold of 0.05 (two‐tailed), controlling the expected proportion of false positive rate at 5%. Additionally, effect sizes were reported to quantify the magnitude of observed between‐ and within‐group differences. For Cohen's d, values of 0.2, 0.5, and 0.8 were considered small, medium, and large effects, respectively [65]. For partial eta squared (η^2^ₚ), values of 0.01, 0.06, and 0.14 were considered small, medium, and large effects, respectively [66]. Statistical analyses were conducted using R version 4.3.1 (R foundation for Statistical Computing) and MATLAB 2014a (Mathworks, Natick, MA), with a two‐tailed statistical significance set at 0.05.

## 3 Results

### 3.1 Demographic Information and Neuropsychological Assessments

**FIGURE 2:**
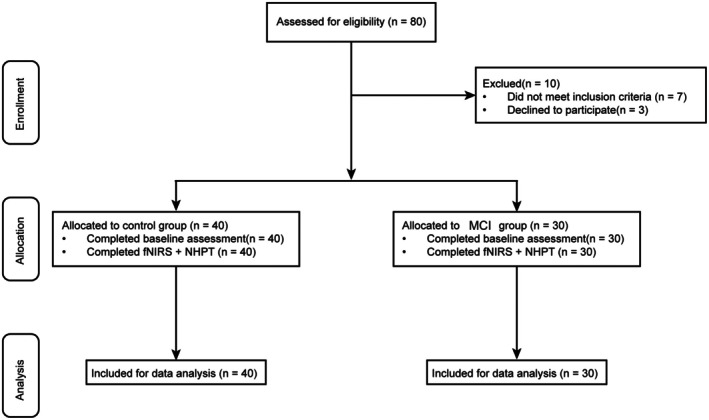
Flowchart illustrating the selection, enrollment, and allocation of participants.

Figure 2 shows the flowchart illustrating participant enrollment. Baseline demographic characteristics and neuropsychological assessments for both HC and MCI groups are summarized in Table 1. No significant differences were observed between the two groups in terms of age, sex, or years of education (*p*‐values ranging from 0.104 to 1.000). However, significant group differences were found across all neuropsychological measures, with the HC group consistently showing greater performance compared to the MCI group (*p*‐values ≤ 0.003). Specifically, the HC group exhibited significantly faster response times on the STT and higher scores on the MMSE, MoCA, AVLT‐H, AFT, and BNT‐C. Functional independence, as assessed by the FAQ scores, was also significantly better in the HC group compared to the MCI group.

**TABLE 1:** Demographic information and neuropsychological assessments of MCI and HC groups.

Variables	HCs (*N* = 40)	MCI (*N* = 30)	*t* or *χ* ^2^or *u* value	*p*
Age (years; Mean ± SD)	64.20 (6.95)	66.90 (6.76)	−1.66^a^	0.104
Sex (F/M)	25/15	19/11	0.00^b^	1.000
Education (years; Mean ± SD)	11.30 (2.70)	10.60 (2.40)	1.13^a^	0.262
Global cognitive function test				
MMSE (score; Mean ± SD)	28.30 (1.57)	26.40 (1.36)	5.64^a^	< 0.001***
MoCA (score; Mean ± SD)	26.30 (2.09)	21.90 (2.52)	8.16^a^	< 0.001***
AVLT‐H				
30‐min delayed recall (score; Median [IQR])	6.00 [2.00, 10.00]	3.00 [0.80, 8.00]	90.00^c^	< 0.001***
Recognition (score; Mean ± SD)	22.40 (1.58)	18.30 (2.99)	9.87^a^	< 0.001***
AFT (score; Mean ± SD)				
Animal fluency score	18.90 (4.62)	14.80 (4.04)	4.55^a^	< 0.001***
BNT‐C (score; Mean ± SD)				
Correct naming score	24.50 (2.85)	19.90 (4.09)	6.54^a^	< 0.001***
STT‐C				
Trail A completion time (seconds; Mean ± SD)	56.40 (13.60)	102.00 (31.20)	−13.56^a^	< 0.001***
Trail B completion time (seconds; Mean ± SD)	141.00 (42.70)	233.00 (65.20)	−17.23^a^	< 0.001***
FAQ (score; Median [IQR])	0.00 [0.00, 2.00]	0.50 [0.00, 8.00]	120.00^c^	0.003**

Abbreviations: AFT, Animal Fluency Test; AVLT‐H, Auditory Verbal Learning Test‐Huashan Version; BNT‐C, Boston Naming Test‐Chinese Version; F, female; FAQ, Functional Activities Questionnaire; IQR, interquartile range; M, male; MMSE, Mini‐Mental State Examination; MoCA, Montreal Cognitive Assessment; SD, standard deviation; STT‐C, Shape Trial Test‐Chinese Version.

^a^ Independent 2‐sample *t* test.

^b^ *χ* ^2^ test.

^c^ Mann–Whitney *U* test.

*p < 0.05, **p < 0.01, ***p < 0.001.

### 3.2 Behavioral Performance on the NHPT

**FIGURE 3:**
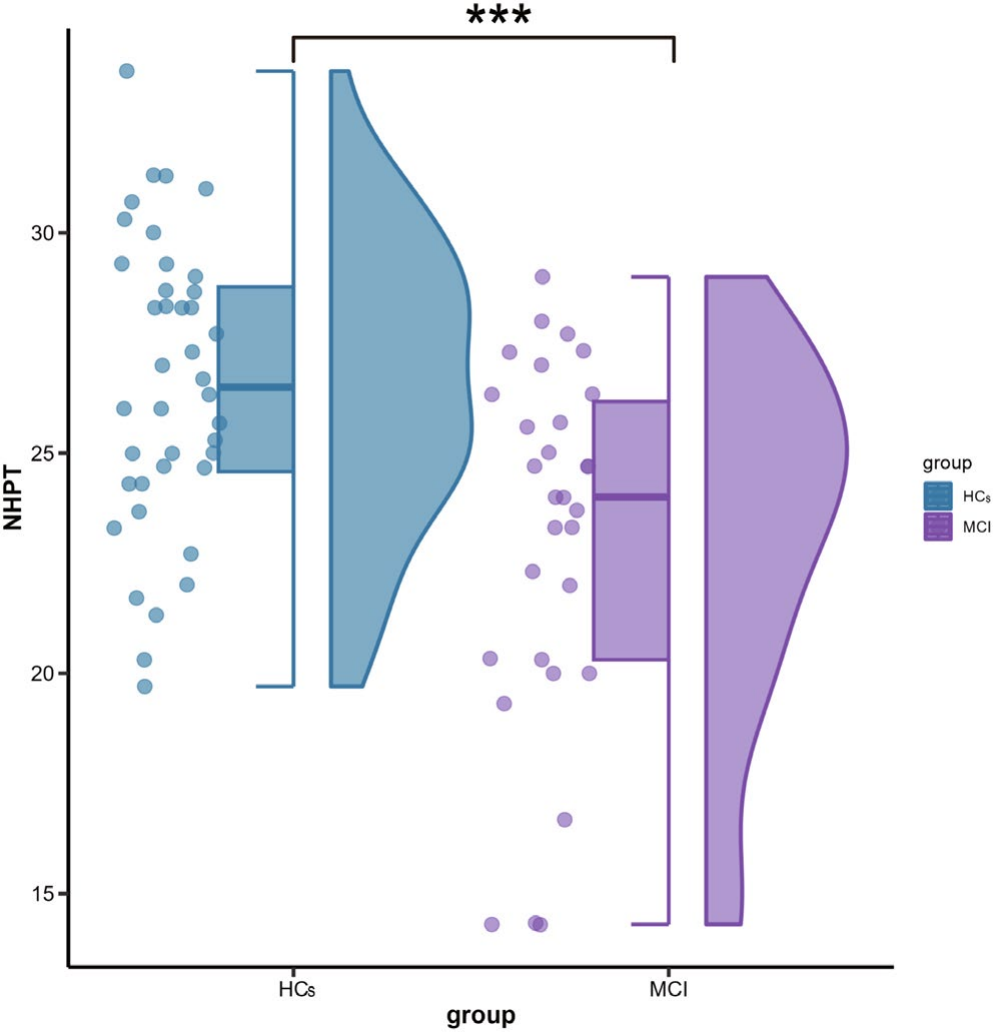
Scatter, box, and violin plots of NHPT scores for individuals with MCI and HCs. Each dot represents an individual score. Box plots represent the medians, interquartile (first and third quartiles), and minimum and maximum values, while scatter and violin plots show the probability density distribution of scores. Asterisks represent the significance levels of group differences (***p < 0.001). Abbreviations: HCs, healthy controls; MCI, mild cognitive impairment; NHPT, Nine‐Hole Peg Test.

The HC group achieved a mean score of 26.55 (SD = 3.29), while the MCI group had a lower mean score of 23.05 (SD = 4.16) (see Figure 3). Independent *t*‐test analysis revealed a significant group difference (*t*(68) = 4.16, *p* < 0.001, *d* = 0.95), indicating that individuals with MCI exhibited reduced manual dexterity compared to HCs.

### 3.3 Hemodynamic Response During the NHPT

**FIGURE 4:**
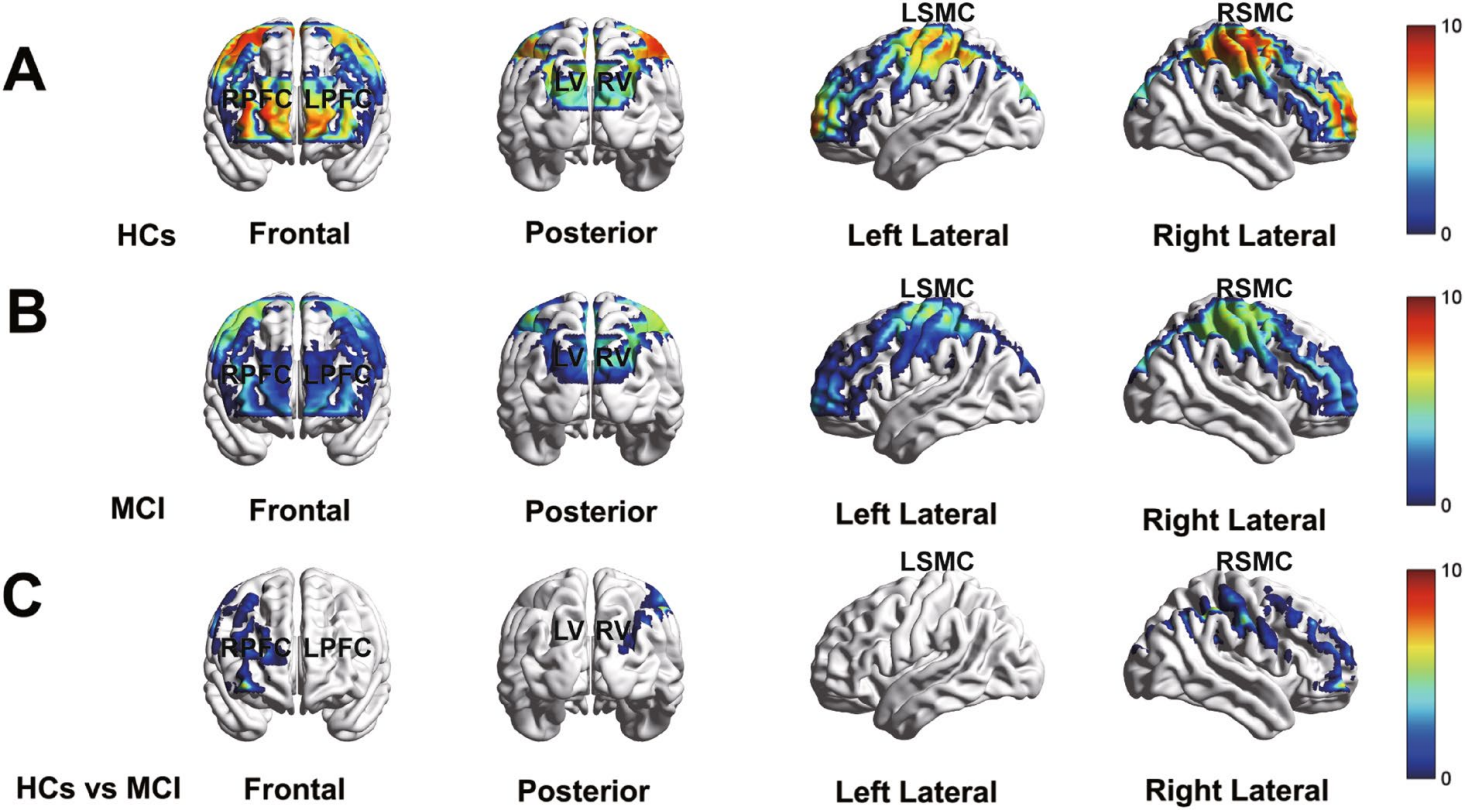
HbO activation maps generated from individual fNIRS channels, illustrating cortical hemodynamic responses during the NHPT. (A) HbO levels in the HC group, showing significant activation relative to the resting period. (B) HbO levels in the MCI group, showing significant activation relative to the resting period. (C) Group comparison of HbO levels between the HC and MCI group, highlighting reduced cortical activation in MCI during fine motor task. Red indicates greater activation, while blue represents reduced activation. Abbreviations: FNIRS, functional near‐infrared spectroscopy; HbO, oxygenated Hemoglobin; HCs, healthy controls; L, left; MCI, mild cognitive impairment; NHPT, Nine‐Hole Peg Test; PFC, prefrontal cortex; R, right; SMC, sensorimotor cortex; VC, visual cortex.

Analysis of task‐related HbO levels revealed significant cortical activation in the bilateral SMC, PFC, and VC in both groups. In the HC group, HbO levels were significantly increased across these regions during the NHPT task compared to the resting period (*t*(39) = 2.50–10.47, *d* = 0.40–1.66, p_adjusted ≤ 0.001 to 0.017; Figure 4A). Similarly, in the MCI group, significant task‐related activation was observed across the same cortical regions (t(29) = 2.55 to 6.97, d = 0.46 to 1.27, p_adjusted ≤ 0.001 to 0.023; Figure 4B). Between‐group comparisons, controlling for age, sex, and years of education, showed that the MCI group exhibited significantly lower HbO responses in the right SMC and PFC during the NHPT task compared to the HC group (*F*(1, 68) = 8.30–12.07, p_adjusted = 0.011–0.031; Figure 4C).

### 3.4 Discriminant Analysis Among the HCs Group and MCI Group

**FIGURE 5:**
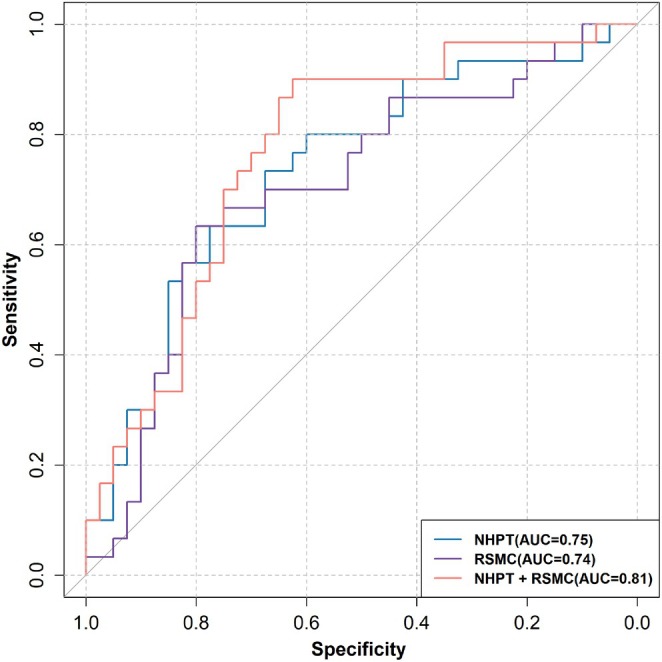
ROC curves illustrating the classification performance of three models in distinguishing MCI from HC: NHPT scores alone, HbO levels in the RSMC alone, and their combined model. The ROC curves show the discriminative power of each model, with the AUC indicating classification accuracy. Abbreviations: AUC, area under the curve; NHPT, Nine‐Hole Peg Test; ROC, receiver operating characteristic; RSMC, right sensorimotor cortex.

To explore the discriminative potential of cortical activation features, three candidate variables were initially identified, and LASSO regression was applied for feature selection. Two key predictors with nonzero coefficients, HbO levels in the right PFC and right SMC, were retained (Figure S1). Stepwise binary logistic regression analysis, adjusted for age, sex, and education, was performed to assess the ability of HbO features and NHPT performance to differentiate MCI from HCs. Seven separate models were tested (Table S7), with the best‐performing model incorporating HbO levels in the right SMC and NHPT scores. This combined model explained 30.1% of the variance (Cox & Snell *R* ^2^ = 0.301) and significantly outperformed models based on single predictors. Receiver operating characteristic (ROC) curve analysis revealed that the combined model achieved an area under the curve (AUC) of 0.81 (95% CI: 0.71–0.91; Figure 5), with an overall classification accuracy of 80.8%. The model achieved a sensitivity of 75% (95% CI: 0.65–0.85) and a specificity of 66.7% (95% CI: 0.50–0.84) at an optimal cut‐off threshold of 0.408. Values above the threshold were classified as MCI, while those below were classified as HCs. Compared with individual predictors, the combined model provided superior predictive performance compared to HbO in the right SMC alone (AUC = 0.742, 95% CI: 0.595–0.889; Sensitivity = 0.78, 95% CI: 0.621–0.929; Specificity = 0.63, 95% CI: 0.452–0.814) and NHPT scores alone (AUC = 0.75, 95% CI: 0.638–0.862; Sensitivity = 0.775, 95% CI: 0.726–0.824; Specificity = 0.567, 95% CI: 0.397–0.737). The performance improvement achieved by the combined model was statistically significant (*χ* ^2^(5) = 25.93, *p* < 0.001). Additionally, model fit was confirmed using the Hosmer‐Lemeshow test (*χ* ^2^(8) = 4.36, *p* = 0.82), indicating that the combined model adequately described the data.

## 4 Discussion

This study employed fNIRS to examine the neural correlates of fine motor deficits associated with MCI. Consistent with our hypothesis, individuals with MCI exhibited significantly impaired manual dexterity on the NHPT, accompanied by reduced hemodynamic responses in the right SMC and PFC compared to HCs. Furthermore, LASSO regression analysis identified HbO levels in the right SMC and PFC as key predictors in distinguishing MCI from HC. Stepwise discriminant analysis showed that a combined model integrating HbO levels in the right SMC with NHPT scores achieved an AUC of 80.8%, significantly improving classification accuracy compared to using either metric alone. These findings provide novel evidence that cortical hemodynamic hypoactivation in the prefrontal and sensorimotor regions contributes to fine motor deficits associated with MCI, suggesting disrupted motor‐cognitive integration in early cognitive decline. Moreover, the high classification accuracy of our combined model highlights the potential of integrating fNIRS‐derived hemodynamic responses with fine motor assessments as a non‐invasive, multimodal framework for MCI detection.

Accumulating evidence suggests that multisensory function and fine motor abilities are closely linked to early cognitive impairment [67], with motor deficits often emerging in individuals at risk of cognitive decline. Given the early manifestation of these impairments in MCI, monitoring fine motor function may provide valuable insights into the progression of cognitive decline and the development of intervention strategies. Our findings revealed significant fine motor deficits in individuals with MCI, as evidenced by poorer performance on the NHPT compared to HCs. This is in line with previous research reporting that individuals with MCI exhibited pronounced bradykinesia and reduced upper limb dexterity in the Purdue Pegboard Test [21]. Similarly, participants with cognitive impairment exhibited significantly lower accuracy and sensitivity than HCs in the Finger Tapping Task [68]. Kinematic handwriting analysis further supports this pattern, showing that individuals with MCI exhibited less regular, slower, less smooth, and less coordinated handwriting movements compared to HCs, with more pronounced deficits observed in AD patients [22, 23]. Collectively, these studies suggest the potential of fine motor assessment as early indicators of cognitive decline.

Beyond behavioral changes, our study revealed significant differences in cortical activation between MCI and HC groups during the NHPT task. Specifically, individuals with MCI exhibited significantly reduced HbO responses in the right PFC compared to HCs. These findings are consistent with prior research showing prefrontal hypoactivation in MCI across various cognitive tasks, including studies showing that individuals with MCI exhibited reduced PFC activation during visual memory span tasks [9] and attenuated median frequency and prolonged positive‐peak latency of EEG responses in the PFC [69]. Unlike these studies that focused on cognitive tasks, our study provides novel insights by investigating cortical activation during a fine motor task, further highlighting the complexity of neurofunctional alterations in MCI. The NHPT, as a precisely timed and coordinated motor task, offers a unique window into prefrontal function in MCI. Particularly, the medial prefrontal cortex (mPFC) has been implicated in the temporal coordination of motor actions across species, regulating movement timing, duration, and synchronization [70]. These functions are critical for tasks requiring precise rhythm and coordination [70], such as the NHPT. Thus, the observed PFC hypoactivation in individuals with MCI may reflect their deficits in motor timing and executive control, suggesting a close relationship between fine motor deficits and disrupted neural activity in the PFC.

Also, individuals with MCI exhibited significantly reduced HbO responses in the right SMC compared to HCs during the NHPT. Similarly, a previous magnetoencephalographic (MEG) study showed that AD patients exhibited a significantly lower somatosensory gating ratio than HCs during a paired‐pulse somatosensory gating task [71]. Our study extends this line of evidence by showing that hemodynamic abnormalities in the SMC can be detected at the early stages of cognitive decline. The SMC plays a crucial role in fine motor execution, facilitating the integration of sequential elements into higher‐order motor representations across various cognitive and sensorimotor domains (e.g., motor, temporal, spatial, numerical, and linguistic processing) [72]. Functionally, the SMC acts as a central hub for motor planning, execution control, and learning of new movement sequences [67]. Additionally, it is involved in precision grip tasks, contributing to fine‐graded timing and force control [73]. Our findings showed that individuals with MCI exhibited reduced SMC activation, coinciding with their poorer fine motor performance compared to HCs. These findings suggest that fine motor deficits in MCI may not merely be a consequence of peripheral motor dysfunction but rather reflect a fundamental disruption in central neural mechanisms. The reduced SMC activation may indicate impaired integration and coordination of motor‐related information, which is critical for executing fine motor tasks such as the NHPT.

Previous research has shown that HbO measurements during visual memory span tasks can effectively differentiate amnestic MCI (aMCI) from HC [9]. Similarly, finger‐tapping paradigms have also shown efficacy in distinguishing MCI from HC [74]. However, these approaches relied on either neurophysiological responses or behavioral performance alone. Our findings address this gap by showing that combining HbO levels in the right SMC with NHPT performance yielded superior classification accuracy (AUC = 80.8%) compared to models based on HbO levels or NHPT performance alone. This synergistic improvement likely reflects the complementary nature of these metrics: while NHPT captures fine motor deficits based on sensorimotor integration, SMC HbO levels index sensorimotor dysfunction critical for motor coordination and execution control. The superior performance of this multimodal prediction model [75] underscores the complexity of MCI pathophysiology, where motor deficits arise from both cortical hypoactivation and disrupted motor–cognitive networks. These results underscore the clinical implication of incorporating fine motor assessments with cortical activation measurements, offering a promising approach for improving detection of subtle functional deficits in MCI.

Finally, this study has several limitations that should be considered. First, the relatively small sample size restricted our ability to perform subgroup analyses within the MCI group, potentially affecting the statistical power and generalizability of our results. Future studies with larger cohorts are needed for a more granular examination of different MCI subtypes and their distinct cortical activation patterns. Second, fNIRS has limited spatial resolution, making it difficult to precisely localize neural activity within specific subregions of the PFC and SMC or subcortical regions such as the cerebellum and basal ganglia. Integrating multimodal neuroimaging approaches could enhance spatial precision to provide a more comprehensive understanding of functional alterations in MCI. Third, our study did not collect additional physiological measures (e.g., heart rate, blood pressure, etc.), which limits our ability to accurately control for systemic physiological artifacts such as scalp blood flow that may influence fNIRS signals independently of cortical activity. Incorporating multimodal physiological monitoring in future research is warranted to improve signal interpretability and allow robust differentiation between neural and extracerebral contributions. Finally, we focused on a single motor task (i.e., the NHPT) in the present study. More benefits can be obtained from incorporating a broader range of motor tasks (e.g., kinematic analyses, force‐tracking tasks, etc.) to further elucidate the relationship between motor dysfunction and cortical activation in MCI.

## 5 Conclusion

In conclusion, the present study found that individuals with MCI exhibited significant neurobehavioral deficits in fine motor control, characterized by poorer performance on the NHPT and reduced cortical activation in both the prefrontal and sensorimotor cortices. Furthermore, the integration of fine motor performance measures with fNIRS‐derived hemodynamic data showed effective classification in distinguishing MCI from HC. These findings advance our understanding of the interplay between motor and cognitive functions in MCI, suggesting that cortical hypoactivation may serve as a key neural contributor to fine motor impairments in early cognitive decline. Such insights provide a promising foundation for developing non‐invasive, cost‐effective tools to identify individuals at risk of dementia.

## Author Contributions

**Han Yang:** writing – original draft, fNIRS imaging processing, statistical analysis, formal analysis, writing – review and editing. **Jing Teng:** writing – original draft, data acquisition and database management, conceptualization, study design. **Yilun Qian:** writing – original draft, data acquisition and database management. **Taicheng Huang:** writing – original draft, writing – review and editing. **Manyu Dong:** data acquisition and database management. **Huanping Wang:** fNIRS imaging processing. **Jie Song:** data acquisition and database management. **Yuxuan Zhang:** fNIRS imaging processing, writing – review and editing. **Mingming Zhang:** fNIRS imaging processing, writing – review and editing. **Hanjun Liu:** writing – original draft, writing – review and editing. **Ying Shen:** conceptualization, study design, writing – original draft, writing – review and editing, safety assessment, supervision, funding acquisition.

## Ethics Statement

The cross‐sectional study protocol adhered to the ethical standards outlined in the Declaration of Helsinki and approved by the Ethics Committee of the First Affiliated Hospital with Nanjing Medical University (No. 2023‐SR‐792).

## Consent

All participants received a detailed explanation of the study procedure and provided written informed consent prior to participation.

## Conflicts of Interest

The authors declare no conflicts of interest.

## Supporting information


**Figure S1:** Feature selection using the least absolute shrinkage and selection operator (LASSO) logistic regression. (A) Cross‐validated binomial deviance plotted against log(λ) using 10‐fold cross‐validation, with dotted vertical lines indicating λ.min (minimum deviance) and λ.1se (1‐standard‐error rule). (B) LASSO coefficient profiles showing shrinkage paths for each variable. Based on 10‐fold cross‐validation and the 1‐standard‐error rule, λ = 0.237 was selected, with two features retained in the final model.

**Table S1:** Detailed overview of fNIRS probe placement, including the exact position of sources and detectors aligned the International10‐20 EEG system. The table also provides the corresponding MNI coordinates (X, Y, Z), the corresponding anatomical landmarks (e.g., dorsolateral prefrontal cortex), and the anatomical specificity of each optode location.

**Table S2:** Mapping of fNIRS channels to regions of interest (ROI) and corresponding Brodmann areas.

**Table S3:** Key functions and parameters used in the Homer2 processing stream for fNIRS data analysis. For detailed description of each function, please refer to the Homer2 documentation (https://www.nitrc.org/projects/homer2).

**Table S4:** Within‐group comparison of HbR levels between task and rest periods in the HC group.

**Table S5:** Within‐group comparison of HbR levels between task and rest periods in the MCI group.

**Table S6:** Between‐group comparison of HbR levels during the NHPT between the HC and MCI groups.

**Table S7:** Performance of predictive models constructed using generalized linear model (GLM) to distinguish individuals with MCI from HCs. Model performance was assessed using 10‐fold cross‐validation. Stepwise discriminant analysis was performed across seven models to identify the most effective combination of features, including NHPT performance, HbO levels, and their integration, for distinguishing between MCI and HC groups.

## Data Availability

The datasets used and/or analyzed in this study are available from the corresponding author upon reasonable request.
