# Supplementary material for: Cortical Hemodynamic Abnormalities Associated With Fine Motor Deficits in Mild Cognitive Impairment
Source: CNS Neurosci Ther. 2025 Jul 28;31(7):e70547. doi: 10.1111/cns.70547 (PMC12304437; doi:10.1111/cns.70547)
Supplement: Supplementary file 1 — Figure S1: Feature selection using the least absolute shrinkage and selection operator (LASSO) logistic regression. (A) Cross‐validated binomial deviance plotted against log(λ) using 10‐fold cross‐validation, with dotted vertical lines indicating λ.min (minimum deviance) and λ.1se (1‐standard‐error rule). (B) LASSO coefficient profiles showing shrinkage paths for each variable. Based on 10‐fold cross‐validation and the 1‐standard‐error rule, λ = 0.237 was selected, with two features retained in the final model. [file CNS-31-e70547-s006.zip › cns70547-sup-0009-DataS1.docx]

**Figure S1.** Feature selection using the least absolute shrinkage and selection operator (LASSO) logistic regression. A) Cross-validated binomial deviance plotted against log(λ) using 10-fold cross-validation, with dotted vertical lines indicating λ.min (minimum deviance) and λ.1se (1-standard-error rule). B) LASSO coefficient profiles showing shrinkage paths for each variable. Based on 10-fold cross-validation and the 1-standard-error rule, λ = 0.237 was selected, with two features retained in the final model.
