# Supplementary material for: Cortical Hemodynamic Abnormalities Associated With Fine Motor Deficits in Mild Cognitive Impairment
Source: CNS Neurosci Ther. 2025 Jul 28;31(7):e70547. doi: 10.1111/cns.70547 (PMC12304437; doi:10.1111/cns.70547)
Supplement: Supplementary file 2 — Table S1: Detailed overview of fNIRS probe placement, including the exact position of sources and detectors aligned the International10‐20 EEG system. The table also provides the corresponding MNI coordinates (X, Y, Z), the corresponding anatomical landmarks (e.g., dorsolateral prefrontal cortex), and the anatomical specificity of each optode location. [file CNS-31-e70547-s001.docx]

**Table S1.** Detailed overview of fNIRS probe placement, including the exact position of sources and detectors aligned the International10-20 EEG system. The table also provides the corresponding MNI coordinates (X, Y, Z), the corresponding anatomical landmarks (e.g., dorsolateral prefrontal cortex), and the anatomical specificity of each optode location.

| **Channel** | **Position** | **Source** | **Detector** | **X(mm)** | **Y(mm)** | **Z(mm)** | **Landmark** | **Specificity(%)** |
| --- | --- | --- | --- | --- | --- | --- | --- | --- |
| 1 | S1-D1 | S1 | D1 | 60 | -11 | 50 | 3 - Primary Somatosensory Cortex | 0.0519 |
|  |  |  |  |  |  |  | 4 - Primary Motor Cortex | 0.063 |
|  |  |  |  |  |  |  | 6 - Pre-Motor and Supplementary Motor Cortex | 0.8852 |
|  |  |  |  |  |  |  |  |  |
| 2 | S1-D6 | S1 | D6 | 59 | -38 | 56 | 1 - Primary Somatosensory Cortex | 0.1095 |
|  |  |  |  |  |  |  | 2 - Primary Somatosensory Cortex | 0.3577 |
|  |  |  |  |  |  |  | 3 - Primary Somatosensory Cortex | 0.0292 |
|  |  |  |  |  |  |  | 4 - Primary Motor Cortex | 0.0182 |
|  |  |  |  |  |  |  | 40 - Supramarginal gyrus part of Wernicke's area | 0.4854 |
|  |  |  |  |  |  |  |  |  |
| 3 | S2-D2 | S2 | D2 | 55 | 37 | -10 | 10 - Frontopolar area | 0.0036 |
|  |  |  |  |  |  |  | 11 - Orbitofrontal area | 0.1127 |
|  |  |  |  |  |  |  | 47 - Inferior prefrontal gyrus | 0.8836 |
|  |  |  |  |  |  |  |  |  |
| 4 | S2-D7 | S2 | D7 | 61 | 25 | 7 | 10 - Frontopolar area | 0.0032 |
|  |  |  |  |  |  |  | 45 - pars triangularis Broca's area | 0.4745 |
|  |  |  |  |  |  |  | 46 - Dorsolateral prefrontal cortex | 0.0892 |
|  |  |  |  |  |  |  | 47 - Inferior prefrontal gyrus | 0.4331 |
|  |  |  |  |  |  |  |  |  |
| 5 | S3-D2 | S3 | D2 | 40 | 63 | -10 | 10 - Frontopolar area | 0.408 |
|  |  |  |  |  |  |  | 11 - Orbitofrontal area | 0.592 |
|  |  |  |  |  |  |  |  |  |
| 6 | S3-D3 | S3 | D3 | 15 | 72 | -9 | 10 - Frontopolar area | 0.2727 |
|  |  |  |  |  |  |  | 11 - Orbitofrontal area | 0.7273 |
|  |  |  |  |  |  |  |  |  |
| 7 | S3-D8 | S3 | D8 | 29 | 69 | 8 | 10 - Frontopolar area | 1 |
|  |  |  |  |  |  |  |  |  |
| 8 | S4-D3 | S4 | D3 | -6 | 72 | -6 | 10 - Frontopolar area | 0.3214 |
|  |  |  |  |  |  |  | 11 - Orbitofrontal area | 0.6786 |
|  |  |  |  |  |  |  |  |  |
| 9 | S4-D4 | S4 | D4 | -30 | 66 | -9 | 10 - Frontopolar area | 0.4896 |
|  |  |  |  |  |  |  | 11 - Orbitofrontal area | 0.5104 |
|  |  |  |  |  |  |  |  |  |
| 10 | S4-D9 | S4 | D9 | -18 | 72 | 9 | 10 - Frontopolar area | 1 |
|  |  |  |  |  |  |  |  |  |
| 11 | S5-D4 | S5 | D4 | -51 | 39 | -13 | 11 - Orbitofrontal area | 0.1282 |
|  |  |  |  |  |  |  | 47 - Inferior prefrontal gyrus | 0.8718 |
|  |  |  |  |  |  |  |  |  |
| 12 | S5-D10 | S5 | D10 | -58 | 22 | 4 | 45 - pars triangularis Broca's area | 0.4552 |
|  |  |  |  |  |  |  | 46 - Dorsolateral prefrontal cortex | 0.0072 |
|  |  |  |  |  |  |  | 47 - Inferior prefrontal gyrus | 0.5376 |
|  |  |  |  |  |  |  |  |  |
| 13 | S6-D5 | S6 | D5 | -58 | -32 | 53 | 1 - Primary Somatosensory Cortex | 0.2008 |
|  |  |  |  |  |  |  | 2 - Primary Somatosensory Cortex | 0.4015 |
|  |  |  |  |  |  |  | 3 - Primary Somatosensory Cortex | 0.0347 |
|  |  |  |  |  |  |  | 4 - Primary Motor Cortex | 0.0656 |
|  |  |  |  |  |  |  | 6 - Pre-Motor and Supplementary Motor Cortex | 0.0463 |
|  |  |  |  |  |  |  | 40 - Supramarginal gyrus part of Wernicke's area | 0.251 |
|  |  |  |  |  |  |  |  |  |
| 14 | S6-D11 | S6 | D11 | -49 | -31 | 64 | 1 - Primary Somatosensory Cortex | 0.2675 |
|  |  |  |  |  |  |  | 2 - Primary Somatosensory Cortex | 0.3333 |
|  |  |  |  |  |  |  | 3 - Primary Somatosensory Cortex | 0.2922 |
|  |  |  |  |  |  |  | 40 - Supramarginal gyrus part of Wernicke's area | 0.107 |
|  |  |  |  |  |  |  |  |  |
| 15 | S7-D1 | S7 | D1 | 50 | -11 | 60 | 3 - Primary Somatosensory Cortex | 0.0954 |
|  |  |  |  |  |  |  | 4 - Primary Motor Cortex | 0.1369 |
|  |  |  |  |  |  |  | 6 - Pre-Motor and Supplementary Motor Cortex | 0.7676 |
|  |  |  |  |  |  |  |  |  |
| 16 | S7-D6 | S7 | D6 | 49 | -35 | 63 | 1 - Primary Somatosensory Cortex | 0.2819 |
|  |  |  |  |  |  |  | 2 - Primary Somatosensory Cortex | 0.2278 |
|  |  |  |  |  |  |  | 3 - Primary Somatosensory Cortex | 0.3475 |
|  |  |  |  |  |  |  | 4 - Primary Motor Cortex | 0.0039 |
|  |  |  |  |  |  |  | 6 - Pre-Motor and Supplementary Motor Cortex | 0.0039 |
|  |  |  |  |  |  |  | 40 - Supramarginal gyrus part of Wernicke's area | 0.1351 |
|  |  |  |  |  |  |  |  |  |
| 17 | S7-D12 | S7 | D12 | 39 | -34 | 71 | 1 - Primary Somatosensory Cortex | 0.1317 |
|  |  |  |  |  |  |  | 2 - Primary Somatosensory Cortex | 0.0285 |
|  |  |  |  |  |  |  | 3 - Primary Somatosensory Cortex | 0.3488 |
|  |  |  |  |  |  |  | 4 - Primary Motor Cortex | 0.3025 |
|  |  |  |  |  |  |  | 6 - Pre-Motor and Supplementary Motor Cortex | 0.1886 |
|  |  |  |  |  |  |  |  |  |
| 18 | S7-D13 | S7 | D13 | 40 | -9 | 68 | 4 - Primary Motor Cortex | 0.0037 |
|  |  |  |  |  |  |  | 6 - Pre-Motor and Supplementary Motor Cortex | 0.9963 |
|  |  |  |  |  |  |  |  |  |
| 19 | S8-D2 | S8 | D2 | 49 | 52 | 6 | 10 - Frontopolar area | 0.8213 |
|  |  |  |  |  |  |  | 46 - Dorsolateral prefrontal cortex | 0.0951 |
|  |  |  |  |  |  |  | 47 - Inferior prefrontal gyrus | 0.0837 |
|  |  |  |  |  |  |  |  |  |
| 20 | S8-D7 | S8 | D7 | 54 | 37 | 25 | 10 - Frontopolar area | 0.1087 |
|  |  |  |  |  |  |  | 45 - pars triangularis Broca's area | 0.0036 |
|  |  |  |  |  |  |  | 46 - Dorsolateral prefrontal cortex | 0.8877 |
|  |  |  |  |  |  |  |  |  |
| 21 | S8-D8 | S8 | D8 | 38 | 58 | 24 | 10 - Frontopolar area | 1 |
|  |  |  |  |  |  |  |  |  |
| 22 | S9-D3 | S9 | D3 | 3 | 70 | 8 | 10 - Frontopolar area | 0.9968 |
|  |  |  |  |  |  |  | 11 - Orbitofrontal area | 0.0032 |
|  |  |  |  |  |  |  |  |  |
| 23 | S9-D8 | S9 | D8 | 17 | 67 | 26 | 10 - Frontopolar area | 1 |
|  |  |  |  |  |  |  |  |  |
| 24 | S9-D9 | S9 | D9 | -9 | 67 | 26 | 10 - Frontopolar area | 1 |
|  |  |  |  |  |  |  |  |  |
| 25 | S10-D4 | S10 | D4 | -45 | 54 | 7 | 10 - Frontopolar area | 0.8128 |
|  |  |  |  |  |  |  | 46 - Dorsolateral prefrontal cortex | 0.1362 |
|  |  |  |  |  |  |  | 47 - Inferior prefrontal gyrus | 0.0511 |
|  |  |  |  |  |  |  |  |  |
| 26 | S10-D9 | S10 | D9 | -31 | 59 | 26 | 10 - Frontopolar area | 1 |
|  |  |  |  |  |  |  |  |  |
| 27 | S10-D10 | S10 | D10 | -52 | 35 | 23 | 45 - pars triangularis Broca's area | 0.0255 |
|  |  |  |  |  |  |  | 46 - Dorsolateral prefrontal cortex | 0.9745 |
|  |  |  |  |  |  |  |  |  |
| 28 | S11-D5 | S11 | D5 | -58 | -7 | 47 | 4 - Primary Motor Cortex | 0.0114 |
|  |  |  |  |  |  |  | 6 - Pre-Motor and Supplementary Motor Cortex | 0.9886 |
|  |  |  |  |  |  |  |  |  |
| 29 | S11-D11 | S11 | D11 | -47 | -6 | 59 | 3 - Primary Somatosensory Cortex | 0.0125 |
|  |  |  |  |  |  |  | 4 - Primary Motor Cortex | 0.0833 |
|  |  |  |  |  |  |  | 6 - Pre-Motor and Supplementary Motor Cortex | 0.9042 |
|  |  |  |  |  |  |  |  |  |
| 30 | S12-D12 | S12 | D12 | 29 | -32 | 75 | 3 - Primary Somatosensory Cortex | 0.178 |
|  |  |  |  |  |  |  | 4 - Primary Motor Cortex | 0.4045 |
|  |  |  |  |  |  |  | 6 - Pre-Motor and Supplementary Motor Cortex | 0.4175 |
|  |  |  |  |  |  |  |  |  |
| 31 | S12-D13 | S12 | D13 | 29 | -9 | 73 | 6 - Pre-Motor and Supplementary Motor Cortex | 1 |
|  |  |  |  |  |  |  |  |  |
| 32 | S13-D11 | S13 | D11 | -39 | -30 | 70 | 1 - Primary Somatosensory Cortex | 0.1619 |
|  |  |  |  |  |  |  | 2 - Primary Somatosensory Cortex | 0.018 |
|  |  |  |  |  |  |  | 3 - Primary Somatosensory Cortex | 0.3885 |
|  |  |  |  |  |  |  | 4 - Primary Motor Cortex | 0.1511 |
|  |  |  |  |  |  |  | 6 - Pre-Motor and Supplementary Motor Cortex | 0.2806 |
|  |  |  |  |  |  |  |  |  |
| 33 | S13-D14 | S13 | D14 | -28 | -27 | 74 | 3 - Primary Somatosensory Cortex | 0.0206 |
|  |  |  |  |  |  |  | 4 - Primary Motor Cortex | 0.3608 |
|  |  |  |  |  |  |  | 6 - Pre-Motor and Supplementary Motor Cortex | 0.6186 |
|  |  |  |  |  |  |  |  |  |
| 34 | S14-D11 | S14 | D11 | -37 | -4 | 66 | 6 - Pre-Motor and Supplementary Motor Cortex | 1 |
|  |  |  |  |  |  |  |  |  |
| 35 | S14-D14 | S14 | D14 | -28 | -4 | 70 | 6 - Pre-Motor and Supplementary Motor Cortex | 1 |
|  |  |  |  |  |  |  |  |  |
| 36 | S15-D15 | S15 | D15 | 25 | -93 | 32 | 19 - V3 | 1 |
|  |  |  |  |  |  |  |  |  |
| 37 | S16-D15 | S16 | D15 | 19 | -104 | 16 | 18 - Visual Association Cortex (V2) | 0.5071 |
|  |  |  |  |  |  |  | 19 - V3 | 0.4929 |
|  |  |  |  |  |  |  |  |  |
| 38 | S17-D15 | S17 | D15 | 13 | -98 | 27 | 19 - V3 | 1 |
|  |  |  |  |  |  |  |  |  |
| 39 | S17-D16 | S17 | D16 | -13 | -100 | 26 | 18 - Visual Association Cortex (V2) | 0.0144 |
|  |  |  |  |  |  |  | 19 - V3 | 0.9856 |
|  |  |  |  |  |  |  |  |  |
| 40 | S18-D16 | S18 | D16 | -20 | -104 | 14 | 18 - Visual Association Cortex (V2) | 0.3905 |
|  |  |  |  |  |  |  | 19 - V3 | 0.6095 |
|  |  |  |  |  |  |  |  |  |
| 41 | S19-D16 | S19 | D16 | -29 | -93 | 28 | 19 - V3 | 1 |
