# Supplementary material for: Cortical Hemodynamic Abnormalities Associated With Fine Motor Deficits in Mild Cognitive Impairment
Source: CNS Neurosci Ther. 2025 Jul 28;31(7):e70547. doi: 10.1111/cns.70547 (PMC12304437; doi:10.1111/cns.70547)
Supplement: Supplementary file 3 — Table S2: Mapping of fNIRS channels to regions of interest (ROI) and corresponding Brodmann areas. [file CNS-31-e70547-s004.docx]

**Table S2.** Mapping of fNIRS channels to regions of interest (ROI) and corresponding Brodmann areas.

| ROI | Brodmann Areas | Channel | |
| --- | --- | --- | --- |
|  |  | Right Hemisphere | Left  Hemisphere |
| Sensorimotor Cortex | \| 1, 2, 3 Primary Somatosensory Cortex \| \| --- \| \|  \| \| 6 Premotor Area / Supplementary Motor Area \| | 2, 16, 17  1, 15, 18, 30, 31 | 13, 14, 32  28, 29, 33, 34, 35 |
| Prefrontal Cortex | \| 10 Frontal Pole \| \| --- \| \| 11 Orbitofrontal Gyrus \| \| 45 Triangular Part of the Inferior Frontal Gyrus (Broca's Area) \| \| 46 Dorsolateral Prefrontal Cortex \| \| 47 Inferior Frontal Gyrus \| | 7, 19, 21, 23  5, 6  4  20  3 | 10, 24, 25, 26  8, 9  12  27  11 |
|  |  |  |  |
| Visual Cortex | 18, 19 Visual Association Cortex | 36, 37, 38 | 39, 40, 41 |

This table denotes the fNIRS channels and their anatomical regions, including the sensorimotor cortex, prefrontal cortex, and visual cortex, categorized by functional sub-regions based on Brodmann areas classifications. Channels are organized by hemisphere (left and right) to indicate specific cortical locations for each ROI.
