# Supplementary material for: Cortical Hemodynamic Abnormalities Associated With Fine Motor Deficits in Mild Cognitive Impairment
Source: CNS Neurosci Ther. 2025 Jul 28;31(7):e70547. doi: 10.1111/cns.70547 (PMC12304437; doi:10.1111/cns.70547)
Supplement: Supplementary file 4 — Table S3: Key functions and parameters used in the Homer2 processing stream for fNIRS data analysis. For detailed description of each function, please refer to the Homer2 documentation (https://www.nitrc.org/projects/homer2). [file CNS-31-e70547-s005.docx]

**Table S3:** Key functions and parameters used in the Homer2 processing stream for fNIRS data analysis. For detailed description of each function, please refer to the Homer2 documentation (https://www.nitrc.org/projects/homer2).

| hmrintensity2OD |  |  |
| --- | --- | --- |
| hmrMotionArtifactByChannel | tMotion | 0.5 |
|  | tMask | 1 |
|  | STDEVthresh | 50 |
|  | AMPthresh | 0.5 |
| hmrMotionCorrectPCA | nSV | 0.8 |
| hmrBandpassFilt | hpf | 0.01 |
|  | lpf | 0.09 |
| hmrOD2Conc | ppf | 6.0 6.0 |

hmrIntensity2OD: convert raw intensity data to optical density;

hmrMotionArtifactByChannel: detect motion artifacts by channel;

tMotion: time window for detecting motion artifacts;

tMask: time window for masking data following motion artifact detection;

STDEVthresh: standard deviation threshold for detecting motion artifacts;

AMPthresh: amplitude threshold for detecting motion artifacts;

hmrMotionCorrectPCA: correct motion artifacts using principal component analysis;

nSV: variance threshold or number of principal components during PCA;

hmrBandpassFilt: apply bandpass filtering to remove physiological noise and slow drift;

hpf: high-pass filter cutoff frequency (Hz);

lpf: low-pass filter cutoff frequency (Hz);

hmrOD2Conc: convert optical density to concentration changes;

ppf: partial pathlength factors for HbO and HbR, respectively.
