# Supplementary material for: Cortical Hemodynamic Abnormalities Associated With Fine Motor Deficits in Mild Cognitive Impairment
Source: CNS Neurosci Ther. 2025 Jul 28;31(7):e70547. doi: 10.1111/cns.70547 (PMC12304437; doi:10.1111/cns.70547)
Supplement: Supplementary file 5 — Table S4: Within‐group comparison of HbR levels between task and rest periods in the HC group. [file CNS-31-e70547-s003.docx]

**Table S4:** Within-group comparison of HbR levels between task and rest periods in the HC group.

| **Channel** | **t** | ***p*_value** | ***p* Value_ FDR** |
| --- | --- | --- | --- |
| channel_1 | -1.01 | 0.32 | 0.52 |
| channel_2 | -2.55 | 0.01 | 0.09 |
| channel_3 | 3.78 | 0.00 | 0.01 |
| channel_4 | 4.39 | 0.00 | 0.00 |
| channel_5 | -4.79 | 0.00 | 0.00 |
| channel_6 | -2.33 | 0.03 | 0.11 |
| channel_7 | -0.17 | 0.86 | 0.89 |
| channel_8 | -1.01 | 0.32 | 0.52 |
| channel_9 | -1.81 | 0.08 | 0.19 |
| channel_10 | 0.56 | 0.58 | 0.66 |
| channel_11 | 3.02 | 0.00 | 0.04 |
| channel_12 | 0.53 | 0.60 | 0.66 |
| channel_13 | -3.01 | 0.00 | 0.04 |
| channel_14 | -1.96 | 0.06 | 0.17 |
| channel_15 | -0.04 | 0.97 | 0.97 |
| channel_16 | -2.38 | 0.02 | 0.11 |
| channel_17 | -1.85 | 0.07 | 0.19 |
| channel_18 | -0.86 | 0.40 | 0.54 |
| channel_19 | 0.32 | 0.75 | 0.81 |
| channel_20 | -1.37 | 0.18 | 0.37 |
| channel_21 | -1.18 | 0.25 | 0.48 |
| channel_22 | -0.21 | 0.84 | 0.88 |
| channel_23 | -0.69 | 0.49 | 0.61 |
| channel_24 | -1.63 | 0.11 | 0.24 |
| channel_25 | -0.54 | 0.59 | 0.66 |
| channel_26 | -1.10 | 0.28 | 0.52 |
| channel_27 | -0.60 | 0.55 | 0.66 |
| channel_28 | -1.91 | 0.06 | 0.17 |
| channel_29 | -2.22 | 0.03 | 0.13 |
| channel_30 | -2.64 | 0.01 | 0.08 |
| channel_31 | -0.84 | 0.40 | 0.54 |
| channel_32 | -2.08 | 0.04 | 0.14 |
| channel_33 | -1.62 | 0.11 | 0.24 |
| channel_34 | -0.82 | 0.42 | 0.54 |
| channel_35 | -0.88 | 0.38 | 0.54 |
| channel_36 | -0.81 | 0.42 | 0.54 |
| channel_37 | 0.83 | 0.41 | 0.54 |
| channel_38 | 1.03 | 0.31 | 0.52 |
| channel_39 | 2.10 | 0.04 | 0.14 |
| channel_40 | 0.82 | 0.42 | 0.54 |
| channel_41 | -2.14 | 0.04 | 0.14 |

channel: fNIRS measurement channel; t: t-statistic from the paired samples t-test; p_value: uncorrected p-value from the t-test; p_value_FDR: p-value corrected for multiple comparisons using the false discovery rate (FDR) method.
