# Supplementary material for: Cortical Hemodynamic Abnormalities Associated With Fine Motor Deficits in Mild Cognitive Impairment
Source: CNS Neurosci Ther. 2025 Jul 28;31(7):e70547. doi: 10.1111/cns.70547 (PMC12304437; doi:10.1111/cns.70547)
Supplement: Supplementary file 6 — Table S5: Within‐group comparison of HbR levels between task and rest periods in the MCI group. [file CNS-31-e70547-s008.docx]

**Table S5:** Within-group comparison of HbR levels between task and rest periods in the MCI group.

| **channel** | **t** | ***p*_value** | ***p*_value_ FDR** |
| --- | --- | --- | --- |
| Channel_1 | 0.04 | 0.97 | 0.98 |
| Channel_2 | -3.63 | 0.00 | 0.02 |
| Channel_3 | 0.93 | 0.36 | 0.63 |
| Channel_4 | 1.60 | 0.12 | 0.33 |
| Channel_5 | -2.41 | 0.02 | 0.12 |
| Channel_6 | 0.75 | 0.46 | 0.76 |
| Channel_7 | 0.06 | 0.95 | 0.98 |
| Channel_8 | 0.91 | 0.37 | 0.63 |
| Channel_9 | -1.35 | 0.19 | 0.43 |
| Channel_10 | 1.93 | 0.06 | 0.22 |
| Channel_11 | 4.64 | 0.00 | 0.00 |
| Channel_12 | 3.23 | 0.00 | 0.04 |
| Channel_13 | -0.91 | 0.37 | 0.63 |
| Channel_14 | -0.47 | 0.64 | 0.83 |
| Channel_15 | -0.47 | 0.64 | 0.83 |
| Channel_16 | -2.52 | 0.02 | 0.10 |
| Channel_17 | -1.88 | 0.07 | 0.22 |
| Channel_18 | -0.59 | 0.56 | 0.83 |
| Channel_19 | 1.08 | 0.29 | 0.57 |
| Channel_20 | 0.35 | 0.73 | 0.91 |
| Channel_21 | 0.03 | 0.98 | 0.98 |
| Channel_22 | -0.48 | 0.64 | 0.83 |
| Channel_23 | -1.13 | 0.27 | 0.55 |
| Channel_24 | -0.05 | 0.96 | 0.98 |
| Channel_25 | 1.47 | 0.15 | 0.39 |
| Channel_26 | 0.30 | 0.77 | 0.93 |
| Channel_27 | 2.68 | 0.01 | 0.10 |
| Channel_28 | 0.08 | 0.93 | 0.98 |
| Channel_29 | -1.77 | 0.09 | 0.26 |
| Channel_30 | -2.52 | 0.02 | 0.10 |
| Channel_31 | -0.15 | 0.88 | 0.98 |
| Channel_32 | -1.36 | 0.18 | 0.43 |
| Channel_33 | -2.31 | 0.03 | 0.13 |
| Channel_34 | -2.04 | 0.05 | 0.21 |
| Channel_35 | -2.72 | 0.01 | 0.10 |
| Channel_36 | 0.59 | 0.56 | 0.83 |
| Channel_37 | -1.27 | 0.21 | 0.46 |
| Channel_38 | -0.49 | 0.63 | 0.83 |
| Channel_39 | -0.21 | 0.83 | 0.98 |
| Channel_40 | -0.51 | 0.61 | 0.83 |
| Channel_41 | -1.90 | 0.07 | 0.22 |

channel: fNIRS measurement channel; t: t-statistic from the paired samples t-test; p_value: uncorrected p-value from the t-test; p_value_FDR: p-value corrected for multiple comparisons using the false discovery rate (FDR) method.
