# Supplementary material for: Cortical Hemodynamic Abnormalities Associated With Fine Motor Deficits in Mild Cognitive Impairment
Source: CNS Neurosci Ther. 2025 Jul 28;31(7):e70547. doi: 10.1111/cns.70547 (PMC12304437; doi:10.1111/cns.70547)
Supplement: Supplementary file 7 — Table S6: Between‐group comparison of HbR levels during the NHPT between the HC and MCI groups. [file CNS-31-e70547-s002.docx]

**Table S6:** Between-group comparison of HbR levels during the NHPT between the HC and MCI groups.

| **Channel** | **F** | ***p*_value** | ***p*_value_FDR** |
| --- | --- | --- | --- |
| Channel_1 | 0.31 | 0.34 | 0.69 |
| Channel_2 | 0.01 | 0.77 | 0.88 |
| Channel_3 | 3.59 | 0.06 | 0.66 |
| Channel_4 | 1.09 | 0.36 | 0.69 |
| Channel_5 | 2.32 | 0.10 | 0.66 |
| Channel_6 | 4.46 | 0.04 | 0.66 |
| Channel_7 | 0.05 | 0.73 | 0.86 |
| Channel_8 | 1.45 | 0.17 | 0.66 |
| Channel_9 | 0.38 | 0.24 | 0.66 |
| Channel_10 | 0.84 | 0.19 | 0.66 |
| Channel_11 | 0.42 | 0.46 | 0.75 |
| Channel_12 | 2.08 | 0.26 | 0.66 |
| Channel_13 | 1.41 | 0.25 | 0.66 |
| Channel_14 | 1.78 | 0.17 | 0.66 |
| Channel_15 | 0.43 | 0.73 | 0.86 |
| Channel_16 | 0.04 | 0.87 | 0.93 |
| Channel_17 | 0.00 | 0.96 | 0.98 |
| Channel_18 | 0.16 | 0.94 | 0.98 |
| Channel_19 | 0.30 | 0.39 | 0.69 |
| Channel_20 | 1.74 | 0.21 | 0.66 |
| Channel_21 | 0.93 | 0.35 | 0.69 |
| Channel_22 | 0.13 | 0.79 | 0.88 |
| Channel_23 | 0.29 | 0.53 | 0.80 |
| Channel_24 | 0.17 | 0.66 | 0.86 |
| Channel_25 | 1.83 | 0.19 | 0.66 |
| Channel_26 | 0.76 | 0.40 | 0.69 |
| Channel_27 | 3.54 | 0.09 | 0.66 |
| Channel_28 | 1.81 | 0.21 | 0.66 |
| Channel_29 | 0.01 | 0.61 | 0.86 |
| Channel_30 | 0.17 | 0.65 | 0.86 |
| Channel_31 | 0.03 | 0.68 | 0.86 |
| Channel_32 | 0.30 | 0.49 | 0.77 |
| Channel_33 | 0.00 | 0.98 | 0.98 |
| Channel_34 | 0.22 | 0.74 | 0.86 |
| Channel_35 | 0.97 | 0.39 | 0.69 |
| Channel_36 | 0.98 | 0.33 | 0.69 |
| Channel_37 | 1.60 | 0.21 | 0.66 |
| Channel_38 | 1.03 | 0.34 | 0.69 |
| Channel_39 | 3.08 | 0.09 | 0.66 |
| Channel_40 | 0.81 | 0.57 | 0.83 |
| Channel_41 | 1.86 | 0.18 | 0.66 |

Channel: fNIRS measurement channel; F: F-statistic from the ANCOVA; p_value: uncorrected p-value associated with the F-test; p_value_FDR: p-value corrected for multiple comparisons using the false discovery rate (FDR) method.
