# Supplementary material for: Cortical Hemodynamic Abnormalities Associated With Fine Motor Deficits in Mild Cognitive Impairment
Source: CNS Neurosci Ther. 2025 Jul 28;31(7):e70547. doi: 10.1111/cns.70547 (PMC12304437; doi:10.1111/cns.70547)
Supplement: Supplementary file 8 — Table S7: Performance of predictive models constructed using generalized linear model (GLM) to distinguish individuals with MCI from HCs. Model performance was assessed using 10‐fold cross‐validation. Stepwise discriminant analysis was performed across seven models to identify the most effective combination of features, including NHPT performance, HbO levels, and their integration, for distinguishing between MCI and HC groups. [file CNS-31-e70547-s007.docx]

**Table S7.** Performance of predictive models constructed using generalized linear model (GLM) to distinguish individuals with MCI from HCs. Model performance was assessed using 10-fold cross-validation. Stepwise discriminant analysis was performed across seven models to identify the most effective combination of features, including NHPT performance, HbO levels, and their integration, for distinguishing between MCI and HC groups.

| Model | AUC | Sensitivity | Specificity |
| --- | --- | --- | --- |
| NHPT | 0.75 (0.638–0.862) | 0.775 (0.726–0.824) | 0.567 (0.397–0.737) |
| RSMC | 0.742 (0.595–0.889) | 0.775 (0.621–0.929) | 0.633 (0.452–0.814) |
| RPFC | 0.683 (0.52–0.846) | 0.7 (0.558–0.842) | 0.533 (0.291–0.776) |
| combine_NHPT_RSMC | 0.808 (0.705–0.912) | 0.75 (0.647–0.853) | 0.667 (0.498–0.835) |
| combine_NHPT_RPFC | 0.733 (0.625–0.842) | 0.775 (0.661–0.889) | 0.6 (0.41–0.79) |
| combine_RSMC_RPFC | 0.708 (0.574–0.842) | 0.675 (0.57–0.78) | 0.6 (0.41–0.79) |
| combine_NHPT_RSMC_RPFC | 0.767 (0.552–0.981) | 0.75 (0.604–0.896) | 0.667 (0.449–0.884) |

**Abbreviations:** NHPT, Nine Hole Peg Test; RSMC, right sensorimotor cortex; RPFC, right prefrontal cortex; AUC, area under the curve.
